# Investigation into the effects of high-Z nano materials in proton therapy

**DOI:** 10.1088/0031-9155/61/12/4537

**Published:** 2016-05-25

**Authors:** R Ahmad, G Royle, A Lourenço, M Schwarz, F Fracchiolla, K Ricketts

**Affiliations:** 1 Division of Surgery & Interventional Science, University College London, London, UK; 2 Department of Medical Physics and Biomedical Engineering, University College London, London, UK; 3 Acoustics and Ionizing Radiation Team, National Physical Laboratory, Teddington, UK; 4 Protontherapy Department, Azienda Provinciale per i Servizi Sanitari (APSS), Trento, Italy; 5 Trento Institute for Fundamental Physics and Applications (TIFPA), National Institute of Nuclear Physics (INFN), Trento, Italy; reem.ahmad.11@ucl.ac.uk

**Keywords:** proton therapy, nanoparticles, range shift

## Abstract

High-Z nano materials have been previously shown to increase the amount of dose deposition within the tumour due to an increase in secondary electrons.

This study evaluates the effects of high-Z nano materials in combination with protons, and the impact of proton energy, nanoparticle material and concentration. These effects were studied *in silico* through Monte Carlo simulation and experimentally through a phantom study, with particular attention to macroscale changes to the Bragg peak in the presence of nanoparticles. Three nanoparticle materials were simulated (gold, silver and platinum) at three concentrations (0.01, 0.1 and 6.5 mg ml^−1^) at two clinical proton energies (60 and 226 MeV). Simulations were verified experimentally using Gafchromic film measurements of gold nanoparticles suspended in water at two available high concentrations (5.5 mg ml^−1^ and 1.1 mg ml^−1^). A significant change to Bragg peak features was evident, where at 226 MeV and 6.5 mg ml^−1^, simulations of gold showed a 4.7 mm longitudinal shift of the distal edge and experimentally at 5.5 mg ml^−1^, a shift of 2.2 mm. Simulations showed this effect to be material dependent, where platinum having the highest physical density caused the greatest shift with increasing concentration. A dose enhancement of 6%  ±  0.05 and 5%  ±  0.15 (60 MeV and 226 MeV, respectively) was evident with gold at 6.5 mg ml^−1^ to water alone, compared to the 21%  ±  0.53 observed experimentally as dose to film with 5.5 mg ml^−1^ of gold nanoparticles suspended in water at 226 MeV. The introduction of nanoparticles has strong potential to enhance dose in proton therapy, however the changes to the Bragg peak distribution that occur with high concentrations need to be accounted for to ensure tumour coverage.

## 1. Introduction

Currently there is a growing interest in the use of high-Z nanoparticles (NPs) in radiotherapy, due to both a predicted and experimentally observed dose enhancement with photon irradiation (Chithrani *et al* 2010, Lechtman *et al* 2013, Taupin *et al* 2015). If targeted to the tumour, high-Z NPs can localise and enhance dose specifically to the tumour and thus giving potential to minimise healthy tissue radiation damage. One of the first studies demonstrating this effect was carried out using gold NPs in mice where a one-year survival of 86% was found with NPs compared to 20% with radiation alone (Hainfeld *et al* 2004).

Few NP studies have been carried out in proton beams, including a number of Monte Carlo simulations. Wälzlein *et al* (2014) conducted a simulation of a nanosphere of controllable material surrounded by water, with proton dose enhancement up to a factor of 2 for gold and platinum at 80 MeV. Another Monte Carlo study conducted by Gao and Zheng (2014) simulated a single gold NP within a water phantom; they concluded that the production of secondary electrons increased with decreasing proton energy, whereas the average kinetic energy of secondary electrons stemming from gold NP interaction increased with proton energy. Kwon *et al* (2015) also simulated a single gold nanoparticle within water. Here however they considered the radial dose distribution due to secondary electrons, where they found that the effect due to gold nanoparticles extended over several micrometers in the longitudinal direction and several nanometers in the radial direction. Lin *et al* (2014) demonstrated a difference in enhancement mechanisms between proton and photon NP-interactions through Monte Carlo simulations, where with protons, the highest enhancement was found closer to the NP. A biological model showed that for protons a higher NP concentration was needed to achieve the same effect compared to photons for extra-nuclear cell-internalised gold NPs (Lin *et al* 2015).

A number of biological studies support the dose enhancement evident in simulations. Kim *et al* (2012) investigated the use of gold and iron nanoparticles in a mouse model at incident proton energy 45 MeV; a one-year survival of 58–100% was found with nanoparticles compared to 11–13% with irradiation alone. Polf *et al* (2011) investigated nanoparticles of diameter 44 nm located in a tissue equivalent phantom at concentration 1 ng/cell. An increase in the effectiveness of tumour cell killing by 15–20% was found at incident proton energy 160 MeV.

As evident from these studies, there is potential for proton dose enhancement from the introduction of nanoparticles into tissue; however, the optimum nanoparticle parameters have not been determined for proton therapy. To summarise, photon studies have demonstrated that the level of enhancement is influenced by the size, concentration, material of nanoparticles and beam energy. It has been shown by Chithrani *et al* (2006) that NP cellular uptake through endocytosis is NP size dependent with an optimum size of 50 nm.

Previous Monte Carlo studies have successfully demonstrated the beneficial effects of nanoparticles on the nanoscale. However, these studies have modelled a single nanoparticle and therefore do not demonstrate the macro scale effects or bulk effect of clinically relevant concentrations of nanoparticles on the Bragg peak (BP) position and shape. Here, we present a nano-film model able to simulate nanoparticle concentrations in a non-computationally expensive manner, with controllable material type and concentration. The simulations investigated three clinically relevant materials, (Gold (Au), Silver (Ag) and Platinum (Pt)), all of which are biocompatible and can therefore be used clinically (Butterworth *et al* 2008, Porcel *et al* 2010, Jain *et al* 2014). Three clinically relevant concentrations were considered (0.01 mg ml^−1^, 0.1 mg ml^−1^ and 6.5 mg ml^−1^) at two proton energies (60 MeV and 226 MeV). These simulations were then validated through an experiment at the Trento Proton Therapy Center, Italy, which had a maximum beam energy of 226 MeV.

## 2. Materials and methods

### 2.1. Monte Carlo simulation

The Monte Carlo simulation toolkit Geant4 version 10.0p02 (Agostinelli *et al* 2003) was used to investigate the effects of high-Z nano-materials using incident protons. Using a similar idea to that of Allen *et al* (1999) where they explored the use of thin foils, we were able to create a geometry that used nano-films. Nano-films were used to limit the computational time for 1D nano-distributions.

Each film had a width and length equal to those of our simulated water phantom, and thickness in dimensions of nanometers. Using this setup, we were able to simulate a nanoparticle concentration by modelling the average inter-nanoparticle distance for the concentration being considered, where this spacing would represent the thickness of water contained between subsequent films. The spacing was given by equation (1),

1 \begin{eqnarray*}r\sim \frac{1}{{{n}^{1/3}}}\end{eqnarray*}

where *r* is the inter-particle spacing and *n* is the particle density. Calculating nano-film numbers in such a manner allowed us to also account for differences in density, where denser materials would have fewer nano-films for the same concentration. With this calculation the average inter-particle distance was approximated, such that the value was divisible into phantom layers 50 nm thick; this was the selected thickness of the nano-film corresponding to the optimum nanoparticle diameter quoted for cellular uptake (Chithrani *et al* 2006). The inter-nano-film distance varied between 0.45 mm at 6.5 mg ml^−1^ to 5.15 mm at 0.01 mg ml^−1^, depending on the concentration and physical density of the material.

**Figure 1.**
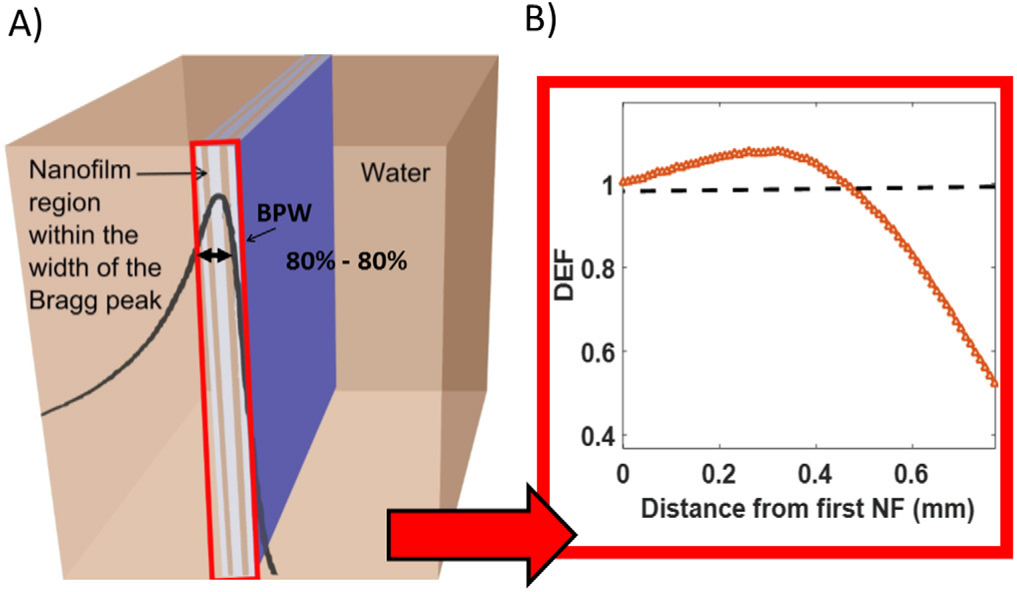
(A) Nano-film simulation model. Nano-films were contained within the 80% to 80% width of the BP within a water phantom. Nano-films had length and width equal to that of the water volume and thickness 50 nm. (B) Enhancement range shows the distance over which the nano-films enhance the dose (DEF  >  1).

Films were placed within the BP width, defined as the distance between the 80% proximal and 80% distal dose (figure 1). A control was performed without nano-films for comparison. For the purposes of this study, two clinically relevant energies were simulated, one at 60 MeV, commonly used for ocular tumours (Damato *et al* 2005), the other at 226 MeV, used predominately for deep-seated tumours. The beam sizes had diameters of 7 mm and 2.7 mm for energies of 60 and 226 MeV respectively, representative of clinical beam sizes. A monoenergetic proton beam was used with 8  ×  10^6^ incident protons, to achieve Poisson statistical errors less than 2%.

The main assumption of the nano-film model was that the nanoscale was simulated in the longitudinal beam direction only. For this assumption to be valid a correction was applied to exclude secondary particles which travelled laterally. Using the energy deposited it was then possible to calculate the dose to water, allowing depth dose plots to be plotted to investigate BP changes. In order to quote energy deposition to water, energy deposited within the nano-films was excluded. This was similarly carried out for the secondary particles recorded within the nano-films.

By using the nano-film model it was possible to investigate many different aspects of dose enhancement and energy distribution: (i) BP width (previously defined) was calculated, related to changes to the Bragg peak shape. Due to changes in shape, measurements were carried out at two points. The first was between the proximal 80% and distal 80% defined as the width of the BP, the other between the proximal 80% and distal 10% demonstrating changes that occur towards the end of range. (ii) Longitudinal shift, related to potential changes in tumour coverage, defined at the end of range, using the depth where the dose had fallen to 0 Gy for each material compared to water. For the 226 MeV simulations a dose value was obtained every 0.1 mm within this region, whereas for 60 MeV this was at every 0.01 mm. (iii) Dose enhancement factor (DEF), related to physical dose and defined as the ratio of the dose to water with nano-films compared to the dose to water alone. DEF was quoted within specific regions; (a) comparing peak dose values of water  +  nano-films to water alone, (b) considering the volume up to the peak dose for water  +  nano-films to water alone. These measures allowed for any changes in the shape of the BP to be taken into account. (iv) Enhancement range, related to BP shape changes and, defined as the distance spanning the beginning of the nano-film region, where the DEF is 1, and ending at the last point where the DEF is 1, (figure 1(B)). This allowed for longitudinal shifts or narrowing of the BP to be quantified. (v) The type of secondary particles produced, to investigate the cause of the predicted enhancement.

### 2.2. Experimental measurements

**Figure 2.**
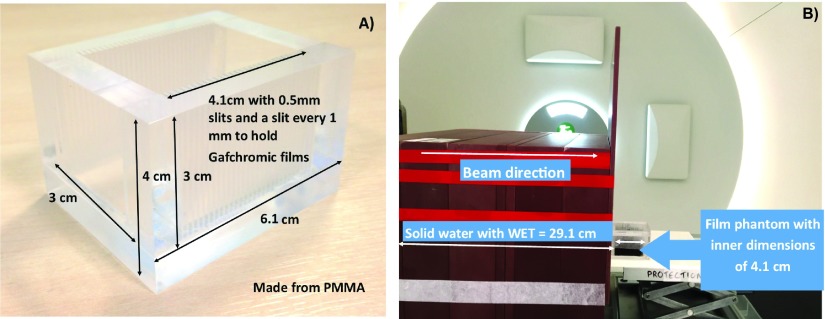
(A) Custom made film phantom able to take measurements 1 mm apart. (B) Setup used for the experiment in Trento comprised of solid water slabs, used to provide sufficient buildup before the film phantom. The water equivalent thickness (WET) of the slabs was 29.1 cm allowing the width of the BP to fall within the film phantom.

To validate our findings, measurements were carried out at the Trento Proton Therapy Center, Italy, using a 226 MeV beam. A phantom was developed to provide i) dose measurement at (ii) sufficient spatial resolution of dose measurement at a range of nanoparticle concentrations. Gafchromic films were used for our measurements, although some have reported an underestimation within the BP region (Reinhardt *et al* 2012). This was not an issue as a relative measurement was needed to compare with and without nanoparticles, and so the overall enhancement effects along with changes to the BP would still be observed. Therefore, EBT3 (Ashland, USA) was used along with a custom made PMMA phantom which could hold Gafchromic film pieces at different depths through the phantom along the beam direction (figure 2).

The phantom had external dimensions of 6.1  ×  5  ×  4 cm^3^ and internal dimensions of 4.1  ×  3  ×  3 cm^3^. Dose measurements were obtained at 1 mm intervals. Each slit, for film placement, was 0.5 mm thick. Films were marked for orientation and labelled for scanning post-irradiation. A concentration of 5.5 and 1.1 mg ml^−1^ of spherical gold nanoparticles with a diameter of 50.7  ±  7.1 nm (nanoComposix, San Diego), suspended in de-ionised water were used to assess the effect of concentration on dose. A concentration of 6.5 mg ml^−1^, equal to the highest simulated concentration, was not commercially available, where this concentration was simulated as it was representative of *in vivo* gold nanoparticle uptake. The concentrations of 5.5 and 1.1 mg ml^−1^ were sufficient as the experiment was used to verify the macro scale changes, rather than a direct quantitative comparison. In order to carry out a direct comparison, additional simulations were carried out, for gold, to demonstrate the simulated effects with concentrations of 5.5 and 1.1 mg ml^−1^.

As shown in figure 2 the setup consisted of several blocks of solid water (Gammex, Middleton, WI) and an entrance window comprised of PMMA, with a total water equivalent thickness (WET) of 29.1 cm, creating a sufficient build-up of dose, allowing the BP width to be contained within the phantom. The main compartment of the film phantom had a lateral depth of 4.1 cm filled with solution of either distilled water or de-ionised water  +  gold nanoparticles of a known concentration. Whilst carrying out the irradiations, the phantom was aligned with the beam such that the width and depth of the BP were in the phantom. This was achieved using a large diameter multi layer ionization chamber, Giraffe (IBA Dosimetry), which allowed us to confirm the WET of our setup.

A beam energy of 226 MeV was used and each depth was irradiated with 20 MU corresponding to a dose range of 6–37 cGy. Gafchromic films were calibrated covering a range from 0–250 cGy, using a clinically used procedure (Reinhardt *et al* 2012). In summary an ion chamber was used to determine the dose delivered to the film, where a calibration curve was produced to convert values of Gafchromic film optical density to known dose. Reference water measurements were carried out initially using Gafchromic films at depths of 0 and 17 cm outside the film phantom and nine measurements in the phantom between 30.3 and 33.2 cm, corresponding to a net peak water dose of 37 cGy, such that a depth dose plot could be obtained. After irradiation each film was contained in a light-tight envelope and kept at room temperature. The film phantom was filled with the two nanoparticle concentrations and irradiated. After 24 h the results were read out using an EPSON desktop scanner, where the pixel values from the red channel and optical density was determined for each measured point. With the depth dose plot, it was possible to determine the DEF for each concentration. The plot was also used to calculate the expected shift of the distal edge, where as before, the end of range was used comparing water  +  gold nanoparticles to water alone.

## 3. Results

### 3.1. Effect on the distribution of energy deposition

Nano-films demonstrated the effects on energy distribution in the longitudinal beam direction, where it was shown that high concentrations caused changes to Bragg peak shape through both a shift in the distal edge and a narrowing of the overall BP width. Depth dose plots were used to show the effect of nano-films on energy deposition.

#### 3.1.1. Simulated longitudinal shift and BP width narrowing.

**Figure 3.**
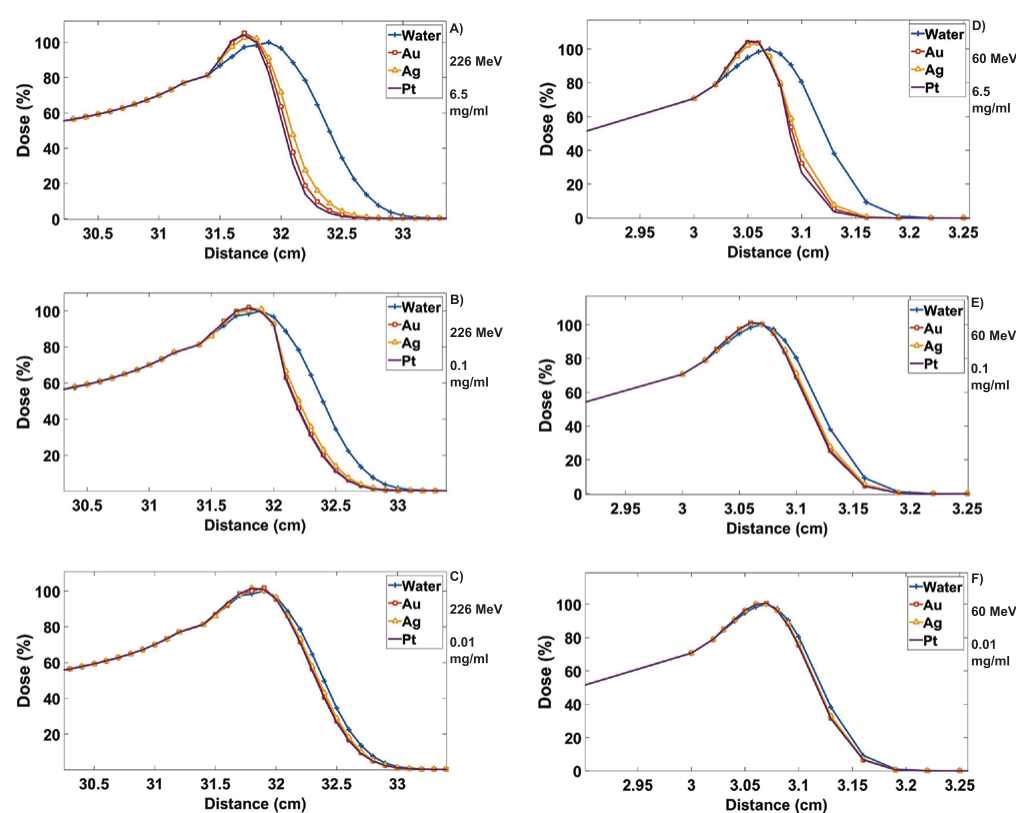
Plots shown of simulated nano-film results, with nano-film regions beginning at depths of 31.31 and 3.02 cm for 226 and 60 MeV, respectively. All materials normalised to the peak water dose, demonstrating the level of enhancement and changes to the BP at 226 MeV for concentrations of (a) 6.5 mg ml^−1^ (b) 0.1 mg ml^−1^ and (c) 0.01 mg ml^−1^ and 60 MeV for (d) 6.5 mg ml^−1^ (e) 0.1 mg ml^−1^ and (f) 0.01 mg ml^−1^.

High concentrations resulted in significant changes to the shape of the BP (figure 3).

The longitudinal shift for both energies are presented in table 1. The highest shift was found to be 5.3 mm with Pt at 226 MeV with a concentration of 6.5 mg ml^−1^.

**Table 1.** Longitudinal shift in the BP for all materials and all concentrations for an energy of 60 and 226 MeV.

Material	Longitudinal shift in the BP (mm)					
	6.5 mg ml^−1^		0.1 mg ml^−1^		0.01 mg ml^−1^	
	60 MeV	226 MeV	60 MeV	226 MeV	60 MeV	226 MeV
Ag	0.34 ± 0.01	3.7 ± 0.1	0.09 ± 0.01	0.9 ± 0.1	0.04 ± 0.01	0.5 ± 0.1
Au	0.40 ± 0.01	4.7 ± 0.1	0.11 ± 0.01	1.3 ± 0.1	0.05 ± 0.01	0.6 ± 0.1
Pt	0.43 ± 0.01	5.3 ± 0.1	0.12 ± 0.01	1.4 ± 0.1	0.06 ± 0.01	0.7 ± 0.1

The BP width had been narrowed due to the nano-films. As narrowing causes changes to the shape of the BP, measurements were carried out at two different points. As before, this increased with material density and concentration, where the results have been summarised in table 2. The greatest level of narrowing can be seen for the 80%–10% BP dose line at 226 MeV, where Pt with a concentration of 6.5 mg ml^−1^ shows a narrowing of approximately 40%. Similarly, for the 80%–80% BP dose line at 60 MeV, Pt also showed the greatest change with 6.5 mg ml^−1^, where a narrowing of approximately 39% was observed.

**Table 2.** Percentage of narrowing in the BP width for all materials and all concentrations at 60 and 226 MeV.

Material	Narrowing from 80%–80% compared to water as percentage difference (%)					
	6.5 mg ml^−1^		0.1 mg ml^−1^		0.01 mg ml^−1^	
	60 MeV	226 MeV	60 MeV	226 MeV	60 MeV	226 MeV
Ag	−31.72 ± 0.78	−24.26 ± 0.53	−12.64 ± 0.25	−13.24 ± 0.29	−6.73 ± 0.13	−10.29 ± 0.22
Au	−36.97 ± 0.91	−27.94 ± 0.62	−14.12 ± 0.28	−25.73 ± 0.57	−8.98 ± 0.17	−14.71 ± 0.32
Pt	−39.38 ± 0.96	−35.29 ± 0.78	−15.27 ± 0.30	−19.12 ± 0.42	−8.17 ± 0.15	−17.65 ± 0.39

Material	Narrowing from 80%–10% compared to water as percentage difference (%)					
	6.5 mg ml^−1^		0.1 mg ml^−1^		0.01 mg ml^−1^	
	60 MeV	226 MeV	60 MeV	226 MeV	60 MeV	226 MeV
Ag	−26.46 ± 0.36	−27.91 ± 0.33	−7.99 ± 0.09	−14.73 ± 0.17	−3.50 ± 0.04	−13.18 ± 0.15
Au	−32.12 ± 0.43	−33.33 ± 0.49	−9.62 ± 0.10	−14.34 ± 0.17	−5.16 ± 0.05	−11.63 ± 0.14
Pt	−35.66 ± 0.48	−40.31 ± 0.53	−10.56 ± 0.12	−12.40 ± 0.15	−5.41 ± 0.06	−10.85 ± 0.13

*Note*: Quoted errors correspond to the error propagation associated with the measured BP widths between the material and water values.

#### 3.1.2. Experimental validation of gold nanoparticle narrowing effects.

**Figure 4.**
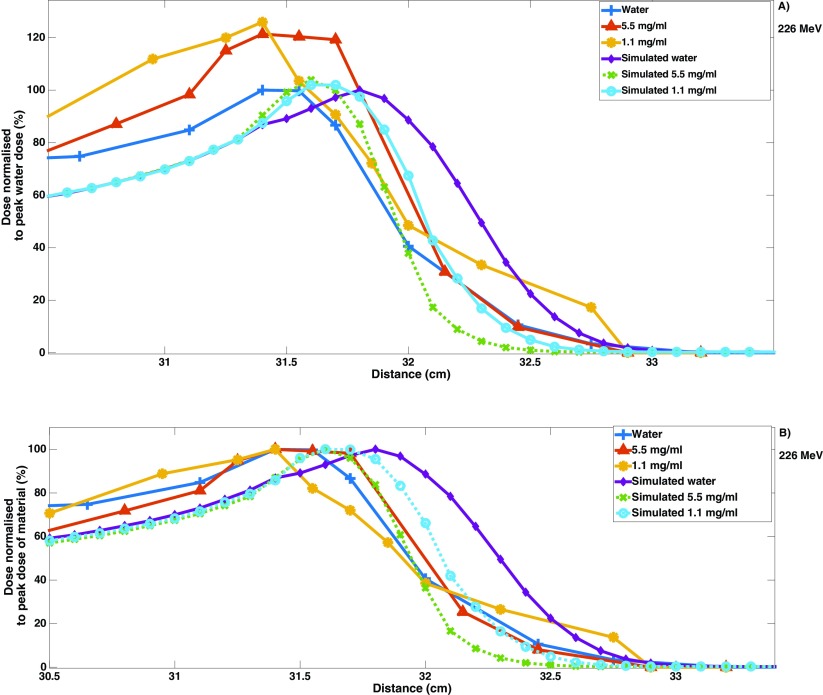
(A) The dose enhancement from both Gafchromic film dose measurement in water and a water–gold nanoparticle solution at concentrations of 5.5 and 1.1 mgAu ml^−1^ and simulated results for these concentrations. (B) Depth dose plot showing the changes to the shape of the BP by normalising each to the respective peak dose.

Figure 4 demonstrates the experimental results of the phantom study, where a 14% narrowing was measured (80–10%), along with a 2.2 mm shift at a concentration of 5.5 mg ml^−1^. This effect was found to be concentration dependent, as no narrowing was observed with 1.1 mg ml^−1^. These two concentrations were also simulated, where the results in figure 4 show a BP shape change with a shift of 4.5 mm and narrowing of approximately 32% (80–10%) for 5.5 mg ml^−1^.

#### 3.1.3. Simulated dose enhancement factor (DEF).

Table 3 summarises the simulated DEF findings, where as expected there was an increase in enhancement with an increase in nano-film concentration and material density. Pt having the highest density, showed DEFs of 1.069 (6.9%) and 1.060 (6%) at 6.5 mg ml^−1^ for 60 and 226 MeV respectively. The difference in DEF between Pt and the other materials for this concentration was less than 2%.

**Table 3.** DEF for peak doses and volume to the peak dose, comparing all materials for all concentrations at energies of 60 and 226 MeV.

Material	DEF of peak dose					
	6.5 mg ml^−1^		0.1 mg ml^−1^		0.01 mg ml^−1^	
	60 MeV	226 MeV	60 MeV	226 MeV	60 MeV	226 MeV
Ag	1.078 ± 6 × 10^−4^	1.027 ± 1.9 × 10^−3^	1.018 ± 3 × 10^−4^	1.016 ± 1.9 × 10^−3^	1.009 ± 2 × 10^−4^	1.012 ± 2.0 × 10^−3^
Au	1.062 ± 4 × 10^−4^	1.053 ± 1.5 × 10^−3^	1.020 ± 3 × 10^−4^	1.020 ± 1.5 × 10^−3^	1.014 ± 5 × 10^−4^	1.017 ± 1.5 × 10^−3^
Pt	1.066 ± 5 × 10^−4^	1.042 ± 1.3 × 10^−3^	1.021 ± 4 × 10^−4^	1.016 ± 1.3 × 10^−3^	1.010 ± 2 × 10^−4^	1.016 ± 1.3 × 10^−3^

Material	Narrowing from 80%–10% compared to water as percentage difference (%)					
	6.5 mg ml^−1^		0.1 mg ml^−1^		0.01 mg ml^−1^	
	60 MeV	226 MeV	60 MeV	226 MeV	60 MeV	226 MeV
Ag	1.050 ± 6 × 10^−4^	1.042 ± 1.7 × 10^−3^	1.015 ± 5 × 10^−4^	1.009 ± 1.0 × 10^−3^	1.008 ± 5 × 10^−4^	1.007 ± 1.4 × 10^−3^
Au	1.063 ± 5 × 10^−4^	1.056 ± 1.5 × 10^−3^	1.019 ± 5 × 10^−4^	1.016 ± 1.3 × 10^−3^	1.010 ± 5 × 10^−4^	1.008 ± 1.4 × 10^−3^
Pt	1.069 ± 5 × 10^−4^	1.060 ± 1.1 × 10^−3^	1.023 ± 4 × 10^−4^	1.018 ± 1.9 × 10^−3^	1.010 ± 5 × 10^−4^	1.010 ± 1.4 × 10^−3^

#### 3.1.4. Simulated enhancement range.

Table 4 demonstrates the enhancement range for the DEF from simulation. It was found that Ag had the highest enhancement range for 60 MeV with 0.58 mm at a concentration of 0.01 mg ml^−1^, compared to 0.46 mm at 6.5 mg ml^−1^. At 226 MeV both Au and Pt offered the highest enhancement range with 5.6 mm at 0.01 mg ml^−1^, compared to 6.5 mg ml^−1^ where Au and Pt were 4.1 mm and 4.0 mm respectively. The main benefit of this measure was to show that although higher concentrations offer a higher enhancement in terms of peak dose value, the range in which they enhance the dose is reduced due to prematurely attenuating the beam. High-Z materials scatter protons through larger angles where an increase in the level of scatter is observed with decreasing concentration due to the larger spacing between subsequent nano-films at low concentrations. As the concentration increases the range over which enhancement occurs is reduced. This is due to the fact that the incident beam has been attenuated by a greater amount of material with the higher concentrations and materials of a higher density.

**Table 4.** Enhancement range for all materials and all concentrations for energies of 60 and 226 MeV.

Material	Enhancement range (mm)					
	0.01 mg ml^−1^		0.1 mg ml^−1^		6.5 mg ml^−1^	
	60 MeV	226 MeV	60 MeV	226 MeV	60 MeV	226 MeV
Ag	0.58	5.2	0.50	5.6	0.46	4.1
Au	0.57	5.6	0.51	5.1	0.45	4.1
Pt	0.52	5.6	0.54	5.1	0.44	4.0

### 3.2. Causes of dose enhancement

**Figure 5.**
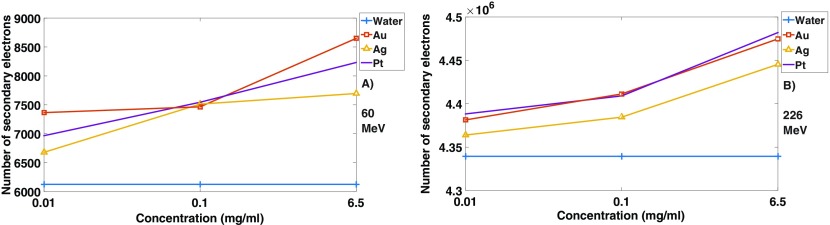
Plot showing the increase in number of secondary electrons with increasing concentration for all materials with (a) 60 MeV beam and (b) 226 MeV.

It is commonly understood within the literature that the main factor causing nanoparticle- induced dose enhancement is the resulting increase in number of secondary electrons (Regulla *et al* 1998, Kwon *et al* 2015). Our model corroborates with this as shown in figure 5, where an increase in the number of secondary electrons could be observed with an increase in nano-film concentration for both energies.

## 4. Discussion

The introduction of high-Z nano materials has been shown to cause a number of changes to the Bragg peak when introduced in proton therapy. The first of these is an increase in the level of dose deposition, demonstrated by the DEF. Pt offered the greatest enhancement at the highest tested nano-film concentration of 6.5 mg ml^−1^ with simulated DEF of 1.069 and 1.060 at 60 and 226 MeV respectively. As expected the level of enhancement increased with concentration, where three orders of magnitude of concentrations were presented. The increase from the lowest to highest concentration for Pt was approximately 6% for 60 MeV and 5% for 226 MeV. The range of enhancement however decreased with increasing concentration, where at 60 MeV (226 MeV) the range for Pt reduced by 15% (29%) from 6.5 mg ml^−1^ to 0.01 mg ml^−1^. From the phantom experiment a DEF of approximately 1.26 was observed with a concentration of 1.1 mg Au ml^−1^ and 1.21 with 5.5 mg Au ml^−1^. This was in disagreement with simulations which showed an increased DEF with concentration; however, it is thought that Gafchromic films were prone to quenching effects which may lead to lower observed DEF at higher concentrations. The DEF observed experimentally was greater than simulation; experiment showed a DEF of 1.21 for gold nanoparticles at 5.5 mg ml^−1^, compared to the simulated DEF of 1.039 for the same concentration.

Importantly, both simulation and measurement found a narrowing of the BP width for nanoparticle  +  water compared to water alone. Simulations showed a narrowing of 23% from the 80%–80% BP dose line and 32% from the 80%–10% BP dose line at 226 MeV with Au at a concentration of 5.5 mg ml^−1^. Experimental findings demonstrated a narrowing of approximately 7% (80%–80%) and 14% (80%–10%) at gold nanoparticle concentration 5.5 mg ml^−1^ and no observable narrowing at 1.1 mg ml^−1^. It is clinically known that high-Z materials cause a level of broadening in the beam, when introduced before the patient. Here however the high-Z materials were located within the width of the BP, towards the end of range, causing a reduction in the level of range straggling and narrowing of the BP width. This resembles the clinical use of nanoparticles in proton beam therapy, where nanoparticles would be expected to fall within the BP width, being targeted and localised within the tumour. As incident protons have a finite amount of energy, their interactions with high-Z materials cause a reduction in the range straggling normally observed with water, where protons reached their end of range closer together due to the barrier caused by the high-Z material. The BP changes were energy dependent; for high energy simulations the effects were more significant due to increased proton range at high energies, where those with longer ranges have more measurable effects. It can be seen that this effect still occurred at the lower energy of 60 MeV. Differences between simulated and measured results were expected due to different set-ups, where simulations did not take beam divergence into account. Another reason for the expected differences was that experimentally the interactions that occur on the nanoscale differ greatly to what can be predicted on the macroscale. Further advancements in Monte Carlo physics lists are needed, for a variety of materials at the nanoscale, in order to improve the accuracy. Importantly, the use of nano-films to simulate nanoparticles within the model innately overestimated the level of change to the BP; in practice nanoparticles would be dispersed in all directions, and the incident proton would not necessarily encounter neighbouring nanoparticles at the frequency of consecutive nano-films. Both BP width narrowing and distal edge shift were overestimated in the simulation, where narrowing was 39% and shift was 51% lower experimentally.

Both simulations and experiment demonstrated a shift in the distal edge. It was found from the simulations that although Pt did not have the highest Z of the materials tested, it had the highest physical density, causing the highest level of change; a 5 mm shift in the distal edge compared to water alone was shown for Pt of concentration 6.5 mg ml^−1^ at 226 MeV. At the same energy, experimental findings demonstrated a 2.2 mm shift in the distal edge for Au at a concentration of 5.5 mg ml^−1^. This shift was expected and is observed clinically, where high density materials are known to cause a range shift in proton beam therapy (Nichiporov *et al* 2011).

In terms of the causes of dose enhancement, the percentage increase in number of secondary electrons with nano-films  +  water compared with water alone was found to be greater at 60 MeV than 226 MeV with an increase of 41% (3%) for Au at concentration 6.5 mg ml^−1^ at 60 MeV (226 MeV). This is in agreement with the findings of Gao and Zheng (2014) which showed a higher number of secondary electrons produced at lower proton beam energies after interactions with gold nanoparticles. This may seem to contradict previous results where a greater shift was observed at higher energies, however the number of secondary electrons for 226 MeV were three orders of magnitude higher than at 60 MeV.

With the level of dose enhancement demonstrated experimentally, it would be beneficial to use gold nanoparticles clinically to increase the level of dose deposition to the tumour. It should also be noted that enhancements presented here demonstrate physical dose enhancement. As demonstrated by other groups with simulated MV photon studies, there is little or no physical dose enhancement predicted for these energies. Experimentally, cellular studies show significant cell kill enhancement and as such biological factors must also be accounted for (Jones *et al* 2010, Lechtman *et al* 2011, Butterworth *et al* 2012).

In terms of the concentrations considered, they are achievable in practice as demonstrated by Hainfeld *et al* (2004). This study quantified *in vivo* gold nanoparticle uptake levels, where an uptake concentration of 6.5 mg Au g^−1^ gold nanoparticles was shown in mice through a single intravenous injection. Just as importantly, a 90% lower concentration was achieved in healthy surrounding tissue. Therefore, the findings presented have the potential to be observed clinically. As such if nanoparticles are used clinically and this localisation is achieved, treatment plans would then need to take these changes in account to ensure an accurate plan. Shifts in the sharp distal edge of the BP have potential to under-dose or miss irradiation of the tumour target (Paganetti 2012). With the introduction of nanoparticles, the cumulative effect on the uncertainties have not been previously evaluated. This study demonstrated experimentally a significant change where in the case of a high gold nanoparticle concentration, a 2.2 mm shift at the end of range should be taken into account. Therefore, with protons, great care must be taken when recommending the use of nanoparticles as the bulk effects on dose distribution must be fully understood, and changes to the BP should be accurately quantified such that treatment plans remain valid. In order to conclude on consequences of changes to the BP, a further study has been proposed, which will incorporate the use of a commercial treatment planning system along with Monte Carlo simulations in order to obtain adequate dose distribution calculations.

## 5. Conclusions

This study demonstrates a flexible working model able to simulate concentrations representative of clinically achievable nanoparticle concentrations, in a non-computationally expensive setup. Compared to other models which consider a single nanoparticle, this model was able to highlight the macro scale effects and investigate changes to the Bragg peak, whilst varying energy, and nanoparticle material and concentration. Both simulation and experiment demonstrated a change in the Bragg peak shape, where a shift of 4.5 mm was simulated for gold with 5.5 mg ml^−1^ compared to 2.2 mm measured experimentally. Differences in simulation to experiment were due to differences in the distribution of gold nanoparticles in solution, as well as what practically occurs at the nanoscale compared to what is predicted on the macroscale. The width of the Bragg peak was shown to narrow in the simulations by approximately 23% compared to 14% from the experiment. Both demonstrated a level of dose enhancement, where experimental findings showed a higher level of 21% compared to simulated 4%. The simulations demonstrated concentration dependent longitudinal shifts in the Bragg peak, which were more evident at higher beam energies due to the longer proton range. At a concentration of 6.5 mg ml^−1^ platinum shifted by 5.3 mm at 226 MeV, compared to 0.43 mm at 60 MeV. These changes were also material dependent, where platinum caused the highest changes, followed by gold then silver, suggesting the effects were dependent on the physical density rather than the atomic number. In terms of the enhancement, simulations showed it to be concentration, material and energy dependent. Overall these results demonstrate the need for quantifying the range of protons in a water to water-nanoparticle setup if nanoparticles are to be used for clinical proton treatments.

## References

[pmbaa2420bib001] Agostinelli S (2003). Geant4 a simulation toolkit. Nucl. Instrum. Methods Phys. Res. A.

[pmbaa2420bib025] Allen X A, Chu J C, Chen W, Zusag T (1999). Dose enhancement by a thin foil of high-Z material: a Monte Carlo study. Med. Phys..

[pmbaa2420bib002] Butterworth K T, McMahon S J, Currell F J, Prise K M (2012). Physical basis and biological mechanisms of gold nanoparticle radiosensitization. Nanoscale.

[pmbaa2420bib003] Butterworth K T, Wyer J A, Brennan-Fournet M, Latimer C J, Shah M B, Currell F J, Hirst D G (2008). Variation of strand break yield for plasmid DNA irradiated with high-Z metal nanoparticles. Radiat. Res..

[pmbaa2420bib004] Chithrani B D, Ghazani A A, Chan W C W (2006). Determining the size and shape dependence of gold nanoparticle uptake into mammalian cells. Nano Lett..

[pmbaa2420bib005] Chithrani D B, Jelveh S, Jalali F, Prooijen M, Allen C, Bristow R G, Hill R P, Jaffray D A (2010). Gold nanoparticles as radiation sensitizers in cancer therapy. Radiat. Res..

[pmbaa2420bib006] Damato B, Kacperek A, Chopra M, Campbell I R, Errington R D (2005). Proton beam radiotherapy of choroidal melanoma: the Liverpool–Clatterbridge experience. Int. J. Radiat. Oncol. Biol. Phys..

[pmbaa2420bib007] Gao J, Zheng Y (2014). Monte Carlo study of secondary electron production from gold nanoparticle in proton beam irradiation. Int. J. Cancer Ther. Oncol..

[pmbaa2420bib008] Hainfeld J F, Slatkin D N, Smilowitz H M (2004). The use of gold nanoparticles to enhance radiotherapy in mice. Phys. Med. Biol..

[pmbaa2420bib009] Jain S (2014). Gold nanoparticle cellular uptake, toxicity and radiosensitisation in hypoxic conditions. Radiother. Oncol..

[pmbaa2420bib010] Jones B L, Krishnan S, Cho S H (2010). Estimation of microscopic dose enhancement factor around gold nanoparticles by Monte Carlo calculations. Med. Phys..

[pmbaa2420bib011] Kim J-K, Seo S-J, Kim H-T, Kim K-H, Chung M-H, Kim K-R, Ye S-J (2012). Enhanced proton treatment in mouse tumors through proton irradiated nanoradiator effects on metallic nanoparticles. Phys. Med. Biol..

[pmbaa2420bib012] Kwon J (2015). Dose distribution of electrons from gold nanoparticles by proton beam irradiation. Int. J. Med. Phys. Clin. Eng. Radiat. Oncol..

[pmbaa2420bib013] Lechtman E, Chattopadhyay N, Cai Z, Mashouf S, Reilly R, Pignol J P (2011). Implications on clinical scenario of gold nanoparticle radiosensitization in regards to photon energy, nanoparticle size, concentration and location. Phys. Med. Biol..

[pmbaa2420bib014] Lechtman E, Mashouf S, Chattopadhyay N, Keller B M, Lai P, Cai Z, Reilly R M, Pignol J P (2013). A Monte Carlo-based model of gold nanoparticle radiosensitization accounting for increased radiobiological effectiveness. Phys. Med. Biol..

[pmbaa2420bib015] Lin Y, McMahon S J, Paganetti H, Schuemann J (2015). Biological modeling of gold nanoparticle enhanced radiotherapy for proton therapy. Phys. Med. Biol..

[pmbaa2420bib016] Lin Y, McMahon S J, Scarpelli M, Paganetti H, Schuemann J (2014). Comparing gold nano-particle enhanced radiotherapy with protons, megavoltage photons and kilovoltage photons: a Monte Carlo simulation. Phys. Med. Biol..

[pmbaa2420bib017] Nichiporov D, Moskvin V, Fanelli L, Das I J (2011). Range shift and dose perturbation with high-density materials in proton beam therapy. Nucl. Instrum. Methods Phys. Res. B.

[pmbaa2420bib018] Paganetti H (2012). Range uncertainties in proton therapy and the role of Monte Carlo simulations. Phys. Med. Biol..

[pmbaa2420bib019] Polf J C, Bronk L F, Driessen W H P, Arap W, Pasqualini R, Gillin M (2011). Enhanced relative biological effectiveness of proton radiotherapy in tumor cells with internalized gold nanoparticles. Appl. Phys. Lett..

[pmbaa2420bib020] Porcel E, Liehn S, Remita H, Usami N, Kobayashi K, Furusawa Y, Le Sech C, Lacombe S (2010). Platinum nanoparticles: a promising material for future cancer therapy?. Nanotechnology.

[pmbaa2420bib021] Regulla D F, Hieber L B, Seidenbusch M (1998). Physical and biological interface dose effects in tissue due to x-ray-induced release of secondary radiation from metallic gold surfaces. Radiat. Res..

[pmbaa2420bib022] Reinhardt S, Hillbrand M, Wilkens J J, Assmann W (2012). Comparison of gafchromic EBT2 and EBT3 films for clinical photon and proton beams. Med. Phys..

[pmbaa2420bib023] Taupin F (2015). Gadolinium nanoparticles and contrast agent as radiation sensitizers. Phys. Med. Biol..

[pmbaa2420bib024] Wälzlein C, Scifoni E, Krämer M, Durante M (2014). Simulations of dose enhancement for heavy atom nanoparticles irradiated by protons. Phys. Med. Biol..

